# An LLM-Based Intelligent Agent and Its Application in Making the Lanolin Saponification Process Greener

**DOI:** 10.3390/ph19020264

**Published:** 2026-02-03

**Authors:** Qinglin Wang, Yu Wang, Xingchu Gong

**Affiliations:** 1 Pharmaceutical Informatics Institute, College of Pharmaceutical Sciences, Zhejiang University, Hangzhou 310058, China; 12419009@zju.edu.cn (Q.W.); 22419174@zju.edu.cn (Y.W.); 2 Jinhua Institute of Zhejiang University, Jinhua 321016, China; 3 National Key Laboratory of Chinese Medicine Modernization, Zhejiang University, Hangzhou 310058, China

**Keywords:** large language model, intelligent agent, green chemistry, lanolin alcohol, microreactor

## Abstract

**Objectives:** The industrial production of lanolin alcohol currently employs batch saponification, which suffers from high energy consumption, prolonged processing time, and excessive solid waste generation, rendering it incompatible with green chemistry principles. This study aimed to develop an efficient, sustainable saponification process by addressing these limitations through integrating large language models (LLMs) with microfluidic technology. **Methods**: An LLM-based intelligent agent called SapoMind (version 1.0) was constructed. SapoMind employs LLMs as its software core and a continuous-flow microreactor as the experimental platform. Its performance was enhanced through supervised fine-tuning. The system enables automated recommendation of saponification process parameters, autonomous experimental design, and automatic execution of experiments. Saponification conditions were automatically optimized considering product quality, energy consumption, material consumption, and time consumption. **Results**: The optimal continuous-flow saponification conditions were determined as 70 °C reaction temperature and 9 min residence time, producing lanolin alcohol complying with European Pharmacopoeia standards. Compared to batch processing, the optimized process reduced carbon emissions by 53% and solid waste by 37%, with the greenness score increasing from 82 to 93. **Conclusions**: This study demonstrates the effectiveness of LLM-driven intelligent agents in optimizing green chemical processes. SapoMind offers significant environmental and operational benefits for lanolin alcohol production.

## 1. Introduction

Medical lanolin alcohol is a natural waxy substance derived from lanolin and is widely used in topical pharmaceutical formulations and cosmetics for the treatment of dry skin and dry eye syndrome [1,2]. Its production typically involves three key steps: saponification of lanolin, water washing, and molecular distillation. According to the European Pharmacopoeia (Ph. Eur. 11.8), medical lanolin alcohol must contain no less than 30% cholesterol and 10–13% lanosterol [3].

Currently, the production of medical lanolin alcohol faces three major challenges. First, quality control is difficult. Precise regulation of both saponification temperature and reaction time is required to ensure the derived lanolin alcohol contains 10–13% lanosterol [1,4]. Second, a large amount of solid waste is generated. To ensure effective saponification, an excess of alkali in the reactor is typically employed in industry [5,6]. Approximately 100 kg of solid residue (primarily waste salts) is produced per ton of lanolin processed, which significantly violates the principles of green chemistry, such as atom economy [7,8,9]. Third, production efficiency is low. Reported saponification time in the literature range from 1.5 to 10 h [10]. Even with pressurized reactions, industrial continuous saponification process requires over 40 min of residence time in the reactor. Consequently, there is an urgent need to optimize the process to achieve efficient and environmentally friendly lanolin saponification.

Continuous-flow reaction in a microchannel is commonly employed for process intensification. When fluids pass through micron-scale channels, they exhibit an ultra-high specific surface area, significantly enhancing mass and heat transfer efficiency [11,12,13]. Compared to conventional stirred-tank reactors, microreactors demonstrate the potential for substantially reducing reaction time while offering superior process controllability and inherent safety characteristics [14,15,16]. The application of this technology to lanolin saponification processes may enable precise control of lanosterol yield, reduce solid-waste generation, and achieve notable time savings.

In recent years, large language models (LLMs) have been increasingly employed by researchers as auxiliary tools to streamline experimental workflows, reduce labor intensity, and enhance research efficiency [17,18,19,20,21,22,23,24]. A representative study by Jensen’s group at MIT successfully demonstrated that integrating machine learning with LLMs enables accurate prediction and accelerated optimization of electrochemical C-H oxidation reactions, utilizing their custom-designed electrocatalytic reactor [18]. Bioko et al. has demonstrated that Coscientist, a GPT-4-powered intelligent agent, was capable of performing web-based literature retrieval, document comprehension, robot control, and automated reaction execution [19]. However, under complex working conditions, the knowledge limitations of LLMs and their inadequate tool scheduling capabilities remained critical challenges that constrained both the efficiency of automated experimental workflows and the reliability of experimental results.

SapoMind, an intelligent agent specifically designed for optimizing continuous lanolin saponification reactions was developed based on LLMs in this research. SapoMind operated through predefined workflows to perform process condition recommendation, experimental design optimization, and automated experimentation, thereby enhancing the tool-scheduling capabilities of LLMs. By incorporating expert knowledge-guided model fine-tuning, we compensated for LLMs’ deficiencies in experimental design and improved the reliability of optimization results. Lanolin saponification was selected as the optimization target for three key reasons: it still relies on inefficient batch reactors in industrial settings, with well-documented process parameters in the literature that provide a solid foundation for further optimization; additionally, its inherent requirements for precise component control and green production perfectly align with the strengths of our LLM-driven workflow. This work aimed to achieve automated condition optimization from batch to continuous processes through the integration of microchannel continuous-flow technology and LLMs, providing a new idea for green chemistry optimization.

## 2. Results

### 2.1. Comparison and Optimization of LLMs

**Figure 1:**
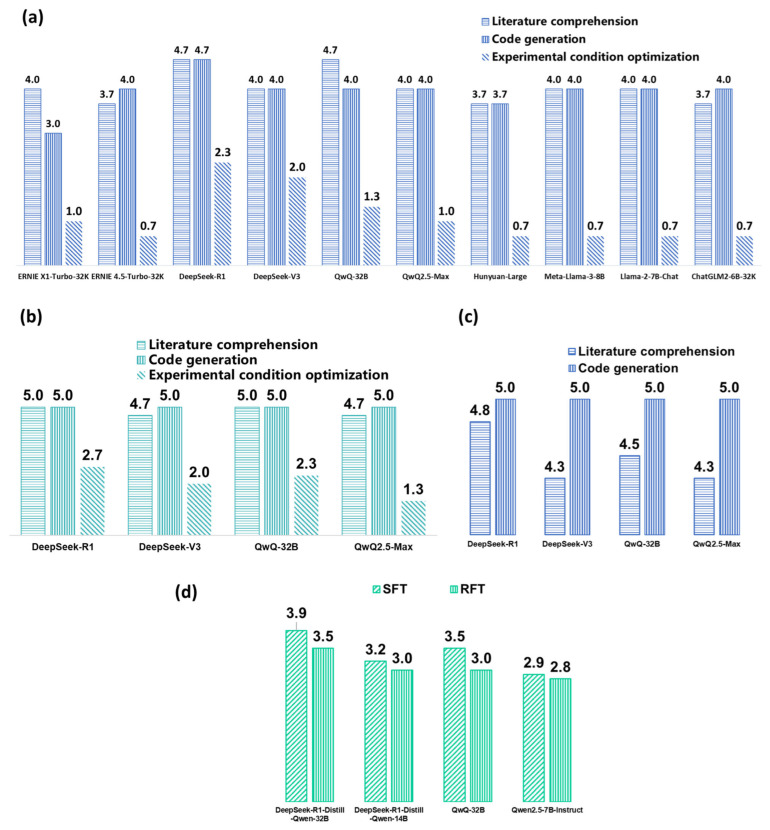
(**a**) Preliminary screening of LLMs. (**b**) Prompt optimization results. (**c**) Validation results of prompt optimization. (**d**) Performance after fine-tuning for the experimental condition optimization task.

After preliminary screening and prompt optimization, DeepSeek-R1 was employed for the literature comprehension and code generation tasks, as it achieved the highest score among the LLMs (Figure 1a–c). Detailed descriptions are provided in Section S1 of Supplementary Material File S1. Representative output results for literature comprehension task and code generation task before and after prompt optimization are shown in Supplementary Material File S1, Figures S1–S4.

To better fulfill the experimental condition optimization task, four LLMs were selected as base models for fine-tuning. The optimized models were evaluated on the constructed dataset, and the results are shown in Figure 1d. After fine-tuning, the models demonstrated a noticeable improvement in their scores for experimental condition optimization. Among them, DeepSeek-R1-Distill-Qwen-32B [25] showed the most significant enhancement. Representative output before and after fine-tuning, are provided in Figures S5–S10 of Supplementary Material File S1. The training/validation loss curve and validation token accuracy of DeepSeek-R1-Distill-Qwen-32B using SFT are shown in Figure S11 of Supplementary Material File S1. The loss curve decreased steadily and eventually stabilized, indicating that the model converged successfully. Meanwhile, the validation token accuracy improved significantly during training, reflecting the model’s effective learning from the data.

Ultimately, the SFT-fine-tuned DeepSeek-R1-Distill-Qwen-32B (referred to as DeepSeek-R1-Distill-Qwen-32B-Data Analysis) was selected for subsequent experiments on experimental condition optimization node.

### 2.2. Automated Execution of Literature-Based Conditions

**Figure 2:**
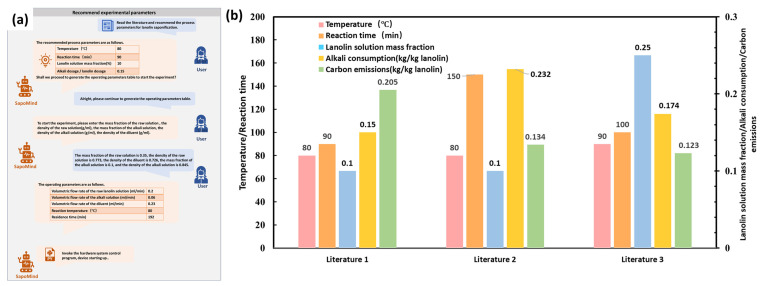
(**a**) Schematic of the dialog interface for automated literature-based condition execution. (**b**) Literature-based conditions and corresponding results.

After users submitted lanolin saponification literature and operational instructions, the intent recognition node identified the user’s objectives. The literature comprehension node and user interaction node generated numerical values for four critical process conditions. After the decision was made to proceed, the reaction solution was prepared according to the questions posed by SapoMind (see Section S3 in Supplementary Material File S1), and the corresponding material properties were measured and provided. SapoMind successfully executed the hardware system through operational-parameter calculation and hardware system operation nodes, based on material properties and key process conditions. The schematic diagram of the question-answering interface is shown in Figure 2a. Detailed interactions from the question-answering process are shown in Supplementary Material File S1, Figure S12.

Three automatic executions under literature-based conditions were conducted. The product collection module automatically gathered the saponified liquid from the outlet, and samples were taken to measure the yields of cholesterol and lanosterol. Results showed the yields of both cholesterol and lanosterol exceeded 96%, indicating nearly complete saponification of cholesterol esters and lanosterol esters in the lanolin. The remaining process conditions and results are shown in Figure 2b. The saponified liquids collected from the three process conditions were combined, followed by water washing and alcohol–acid separation to prepare wool alcohol. The obtained product contained 32.8% cholesterol and 18.7% lanosterol. While the cholesterol content met the requirements of the European Pharmacopoeia, the lanosterol content did not. Further optimization is therefore required based on these results.

### 2.3. Automated Experimental Design and Execution

After the user input the three sets of experimental conditions, results and corresponding instructions into SapoMind, the intent recognition node identified the user’s purpose. Through the experimental condition optimization node and the DSD table generation node, SapoMind determined the scope for experimental design, generated a recommended experimental-design table, and output it. After the decision was made to proceed, the corresponding material properties were provided. Based on the material properties and experimental design conditions, SapoMind automatically executed the hardware system via the operational-parameter calculation node and the hardware operation node.

**Figure 3:**
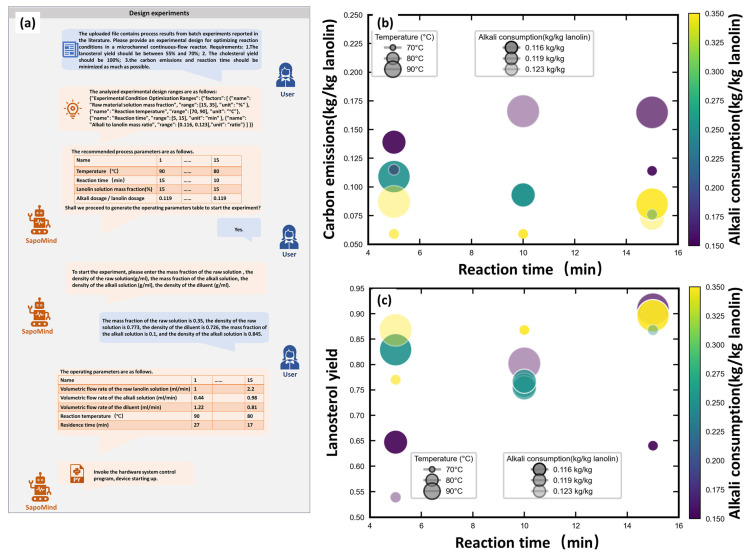
(**a**) Schematic of the dialogue interface for automated experimental design and execution. (**b**) The relationship between lanosterol yield and process conditions. (**c**) The relationship between carbon emissions and process conditions.

A schematic diagram of the dialogue interface for automated experimental design and execution is shown in Figure 3a, and the complete interaction details are provided in Supplementary Material File S1, Figures S13 and S14. During the experiment, a temperature sensor probe continuously monitored the oil bath temperature. Once the deviation between the setpoint and the measured temperature was less than 1 °C, the experiment automatically commenced. To minimize the number of temperature transitions during the process, the experiments were executed in ascending order of temperature.

The entire operation lasted 256 min and automatically completed 15 experiments. The product collection module automatically gathered the samples. After the reaction, the saponified liquid was analyzed for lanosterol and cholesterol yields. The results showed that all cholesterol esters were completely saponified under all experimental conditions, while lanosterol yields varied between 53% and 91%. The relationship between lanosterol yield and process conditions is presented in Figure 3b, and the relationship between carbon emissions and process conditions is shown in Figure 3c.

### 2.4. Modeling and Optimization

**Figure 4:**
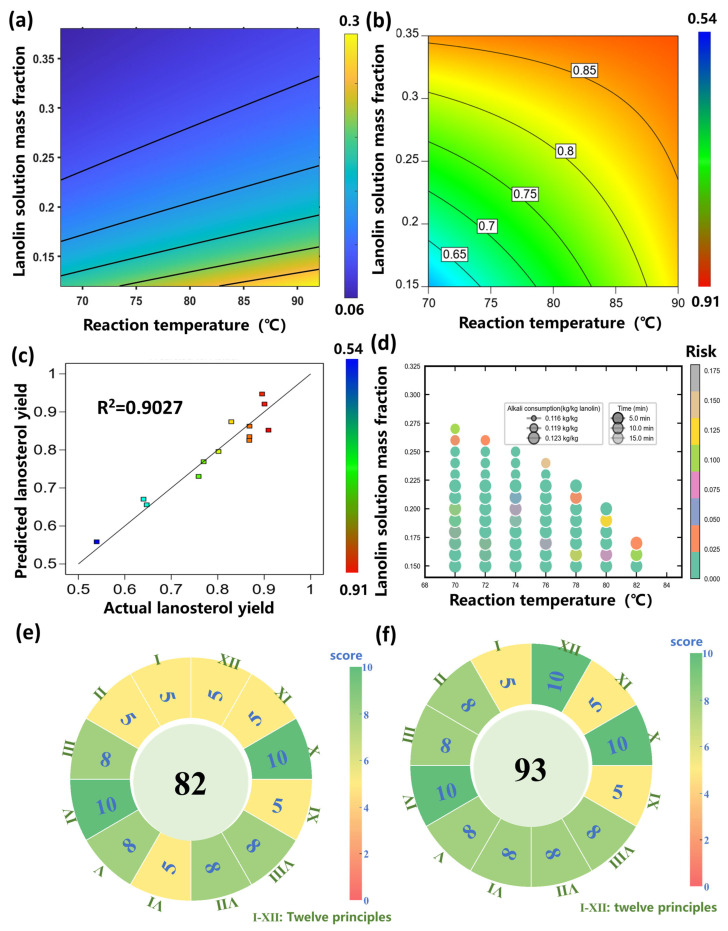
(**a**) Contour plot of carbon emissions, with the color bar indicating the carbon emission value. (**b**) Contour plot of lanosterol yield, with the color bar indicating the yield value. (**c**) Predicted and actual values of lanosterol yield, with the color bar indicating the yield value. (**d**) Risk analysis of optimal conditions, with the color bar indicating the risk value. (**e**) Green score before optimization, with the color bar indicating the score. (**f**) Green score after optimization, with the color bar indicating the score.

The calculation formula for carbon emissions per unit of processed lanolin was derived from the “Guidelines for Accounting Methods and Reporting of Greenhouse Gas Emissions from Chemical Production Enterprises in China” [26], with detailed expressions provided in Section 4.5.1 (Equations (6)–(8)). To illustrate the effects of reaction temperature and the mass fraction of lanolin solution on carbon emission, the reaction time was fixed at 10 min, and the alkali consumption at 0.119 kg/kg lanolin. Figure 4a depicts the variation in carbon emission (ranging from 0.06 to 0.3 kg/kg lanolin) with changes in reaction temperature and the mass fraction of lanolin solution. Carbon emissions increased with higher reaction temperatures and lower lanolin mass fractions. Consequently, to minimize carbon emissions, it is necessary to maintain the lowest feasible reaction temperature and the highest possible lanolin mass fraction.

A quadratic model correlating process conditions with lanosterol yield was developed. The model was simplified via backward stepwise regression with the significance level set at 0.10, and the detailed expression is provided in Section 4.5.2 (Equation (9)). The model fit the data well, with R^2^ = 0.9027 and Adjusted R^2^ = 0.8540, indicating its capability to explain most data variability (Figure 4b). The model has a coefficient of variation of 5.65% and a standard deviation of 0.045, with its ANOVA F-values and *p*-values presented in Table 1. Both the model and all its terms are statistically significant. The model’s significance was confirmed by a *p*-value < 0.001. The contour plot of temperature and lanolin solution mass fraction derived from the model is shown in Figure 4c. The lanosterol yield increased with higher temperature and higher lanolin solution mass fraction. Within the experimental parameter range of this study (alkali dosage: 0.116–0.123 kg/kg lanolin), the effect of alkali dosage on lanosterol yield is not significant. Therefore, in the subsequent optimization process, the alkali dosage was set to the minimum level to reduce solid waste generation.

**Figure 5:**
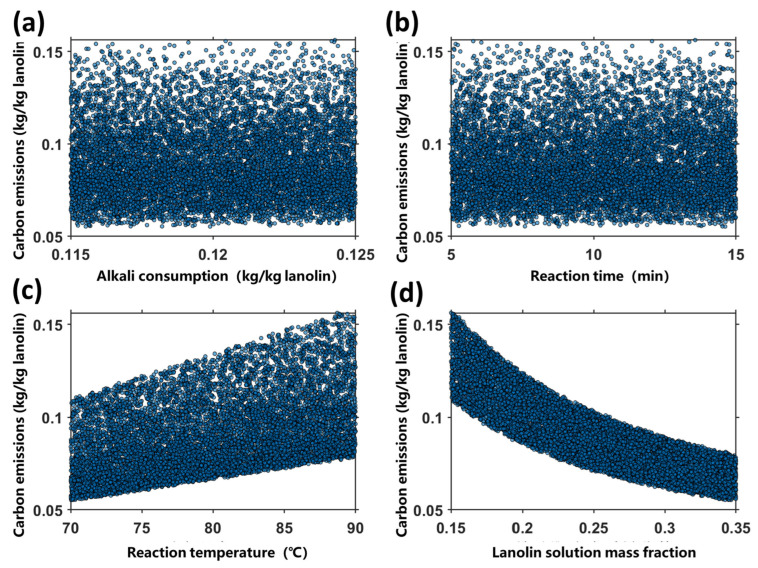
Scatter plots between process parameters and carbon emissions. (**a**) Alkali consumption. (**b**) Reaction time. (**c**) Reaction temperature. (**d**) Lanolin solution mass fraction.

The results of the sensitivity analysis for carbon emissions are presented in Table 2, and the scatter plots between process parameters and carbon emissions are shown in Figure 5. The sensitivity analysis results indicate that the core influencing factors of carbon emissions are the mass fraction of lanolin solution and reaction temperature, while carbon emissions are insensitive to fluctuations in alkali dosage and reaction time. This demonstrates that prioritizing the regulation of the mass fraction of lanolin solution and reaction temperature can achieve the goal of carbon emission reduction with higher efficiency.

The risk quantification method combining the exhaustive method with Monte Carlo simulation was employed to identify robust process conditions. To ensure that the final lanolin alcohol product contained 10–13% lanosterol, the lanosterol yield was systematically constrained within the range of 55–70%. The resulting risk analysis is shown in Figure 4d.

When selecting the optimal process conditions, lanosterol yield, waste generation, process efficiency and carbon emissions were considered in this sequential order. The conditions satisfying the yield requirement are presented in Figure 4d. Waste generation was then optimized: given that alkali dosage dictates waste generation in the saponification reaction, the alkali dosage was set to the minimum value of 0.116 kg/kg lanolin. Process efficiency was subsequently optimized—since shorter reaction times yield higher efficiency, the minimum feasible reaction time within the process design space (5 min) was selected. Finally, carbon emissions were optimized, with higher mass fractions of lanolin solution and lower reaction temperatures resulting in reduced carbon emissions. The reaction temperature was fixed at 70 °C, the minimum value in the design space. For the mass fraction of lanolin solution, risk mitigation was taken into account; for process robustness, it was set at a relatively high value of 0.25.

The specific validation conditions are detailed in Table 3. The predicted value of lanosterol yield was 0.67, while the measured value was 0.67 ± 0.025 (*n* = 3), showing good agreement between the predicted and actual results. The carbon emissions (kg/kg lanolin) were 0.072. After aqueous washing and alcohol–acid separation of the saponification solution, the prepared lanolin alcohol contained 30.5% cholesterol and 11.1% lanosterol, both meeting the requirements of European Pharmacopoeia.

## 3. Discussion

### 3.1. Greenness Assessment

The twelve principles of green chemistry, proposed by Yale University’s Anastas and Warner in 1998, significantly advanced sustainable manufacturing. In 2020, Anastas further articulated twelve defining characteristics for future chemical industries [27], emphasizing atom economy, solvent reduction, and waste minimization in production processes. Implementing green chemistry principles not only reduces ecological risks but also enhances corporate competitiveness through resource efficiency while ensuring occupational and public health safety [28,29,30]. In this study, four optimization indicators evaluated saponification reaction performance from integrated perspectives of product quality, process efficiency, and environmental impact; the obtained results are more aligned with the characteristics of future chemistry.

Compared to the batch production of the saponification process, the optimized process achieved a 53% reduction in carbon emissions and a 37% decrease in solid-waste generation, with a reaction time of 5 min and a total residence time of 9 min.

In this study, a greenness scoring system based on the 12 Principles of Green Chemistry was adopted, with a full score of 10 points for each principle and a total score of 120 points [31]. This scoring approach offers two key advantages: it enables a clear comparison of the changes in greenness scores before and after process optimization against each of the 12 Principles of Green Chemistry, and it also provides a clear direction for the further optimization of the process. After optimization, the consumption of alkali was reduced and the atom economy was improved, leading to a 3-point increase in the score for Principle II (Atom Economy). Additionally, the parameter optimization resulted in lower carbon emissions and reduced energy consumption during the saponification reaction, thus raising the score for Principle VI by 3 points. The adoption of a microreactor has endowed the process with inherent safety, resulting in a perfect score of 10 points for Principle XII. Overall, the total greenness score increased from 82 to 93 points(Figure 4e,f), demonstrating a significant enhancement in greenness.

The correspondence between each scoring item and the relevant Principles of Green Chemistry is provided in Table S5 of the Supplementary Material File S1.

The results of the literature comparison are presented in Table 4. Compared with the findings reported in previous studies, the alkali consumption, reaction temperature, and mass fraction of the lanolin solution in this research are all at moderate levels. However, the reaction time is significantly shortened, leading to a substantial improvement in reaction efficiency. The results under the optimized conditions showed good alignment with the forward-looking characteristics of sustainable development in the chemical industry.

### 3.2. Advantages and Limitations of SapoMind

This study demonstrated the potential of LLM-based agents in automated and green process optimization through predefined workflows and model fine-tuning. The approach reduces manual labor and significantly enhances the sustainability of the saponification process.

Compared with existing automated microreactor systems, the main innovations of SapoMind proposed in this research lie in the following two aspects:

First, it supports a natural language-driven hardware and software system, significantly lowering the learning threshold. Currently, mainstream AI-assisted experimental platforms have achieved remarkable progress in their respective research fields—for instance, the self-driving laboratory (SDL) platform for enzymatic reactions developed by Sebastian Putz et al. [34], the rapid rough screening system for nanomedicine formulations based on liquid-handling robots created by Zeqing Bao et al. [35], and the automated synthesis and optimization platform for perovskite nanocrystals (PNCs) designed by Yixuan Chen et al. [36]. While these platforms have made distinctive breakthroughs, their workflows are relatively rigid, and they lack the ability to autonomously parse unstructured literature and extract process knowledge, leaving room for improvement in terms of usability. In contrast, SapoMind, proposed in this paper, supports natural language dialogue and system control, with simple and user-friendly operation that greatly reduces the user’s learning cost.

Second, it effectively shortens the experimental cycle. Currently, the core self-optimization algorithm for various self-driving laboratory platforms is mainly Bayesian optimization. For example, Clarissa Y. P. Wilding et al. [37] applied it to the optimization of RAFT polymerization to improve efficiency and sustainability, while Tao Song et al. [38] integrated this algorithm into multi-agent-driven robots to discover high-performance metal–organic high-entropy catalysts. However, Bayesian optimization requires researchers to determine the initial exploration space based on prior knowledge or study conditions, which demands a strong professional background. Additionally, the large number of parameters leads to long optimization times, and improper setting of the initial space may result in outcomes deviating from expectations. In contrast, SapoMind, developed in this paper, can accurately define the scope of experimental optimization through supervised fine-tuning with customized datasets. Meanwhile, it leverages large models to achieve automatic literature interpretation, saving manual-literature-review time, and combines experimental design to efficiently find optimal conditions, thereby significantly shortening the experimental cycle.

However, the developed agent SapoMind still exhibits the following limitations:

First, the dataset for fine-tuning primarily focuses on the key chemical reaction parameters (i.e., reaction temperature, material concentration, reaction time, and reactant ratio) and also encompasses the characteristics of reaction equipment conversion from batch to microchannel continuous-flow mode. For target reactions whose core influencing parameters fall outside the aforementioned range (e.g., reaction systems highly sensitive to reaction pressure or catalyst dosage), the generalization ability of the LLM model developed in this study is expected to decline significantly. In subsequent research, we will further expand experimental optimization datasets across diverse process types to enhance the model’s generalization performance. Current experiments are limited to lanolin saponification, and the generalization capability to other reaction systems needs to be validated in the future.

Second, it should be noted that cloud-based deployment of the agent incurs relatively high costs, with fees dynamically fluctuating based on actual call volumes—this makes the approach more suitable for short-term use in the laboratory. Considering the core requirement for data security in industrial production and to significantly reduce long-term operational costs, on-site deployment of the agent is a more optimal option for practical industrial applications.

Thirdly, real-time monitoring has not yet been implemented for SapoMind in this study. To address this limitation, online chromatographic, mass spectrometric or spectroscopic equipment can be added at the outlet of the microreactor in future work, enabling closed-loop optimization based on real-time detection results.

## 4. Materials and Methods

### 4.1. Reagents and Materials

The cholesterol reference standard (purity ≥ 99%, batch no. H2229748) was purchased from Shanghai Aladdin Biochemical Technology Co., Ltd. (Shanghai, China). Acetonitrile (HPLC grade) was obtained from Anhui Tiandi High-Purity Solvent Co., Ltd. (Anqing, China). n-Octane (analytical grade) and n-butanol (analytical grade) were both sourced from Shanghai Aladdin Biochemical Technology Co., Ltd. (Shanghai, China). Potassium hydroxide (analytical grade) was acquired from Sinopharm Chemical Reagent Co., Ltd. (Shanghai, China). Deionized water was prepared using a water purification system (Milli-Q, Millipore, Darmstadt, Germany). Crude lanolin (batch no. AMCZ-B) was provided by Jiangxi Nowi Biotechnology Co., Ltd. (Ji’an, China).

### 4.2. SapoMind Construction

#### 4.2.1. Hardware System

**Figure 6:**
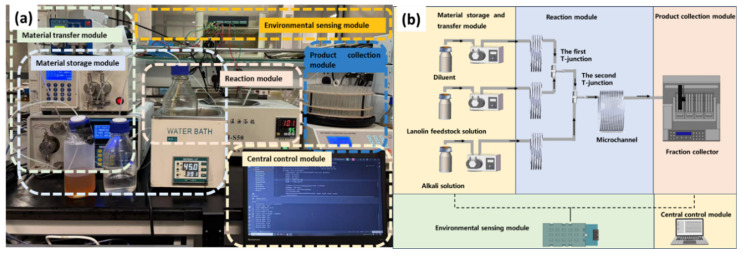
Hardware system. (**a**) Photograph. (**b**) Schematic diagram. Arrows indicate the flow direction of the fluid.

The hardware system comprises five functional modules: (1) material storage and transfer module, (2) reaction module, (3) environmental sensing module, (4) product collection module, and (5) central control module, as illustrated in Figure 6.

The material storage and delivery module is equipped with storage bottles, a water bath (Model DF-101SA, Hangzhou Huichuang Instruments & Equipment Co., Ltd., Hangzhou, China) for maintaining lanolin feedstock solution temperature, and three constant-flux pumps including a lanolin feedstock solution transfer pump (Model MP1006C, Shanghai Sanwei Scientific Instruments Co., Ltd., Shanghai, China), an alkali solution transfer pump (Model 2PB-00C, Beijing Xingda Technology Development Co., Ltd., Beijing, China), and a diluent transfer pump (Model Y-600, Beijing Xiangyue Huanyu Technology Development Co., Ltd., Beijing, China). The reaction module consists of two T-junctions (1 mm inner diameter, 316 stainless steel), an oil bath (Model HH-50, Hangzhou Baoheng Thermostat Technology Co., Ltd., Hangzhou, China), a polytetrafluoroethylene (PTFE) coil (1 mm inner diameter, 50 m length) and a reaction temperature sensor (Model DL10B-M485-V24-D1, Guangzhou Dalin Electronic Technology Co., Ltd., Guangzhou, China). The first T-junction is used for mixing the lanolin feedstock solution with the diluent to form a lanolin solution. The second T-junction is used for mixing the alkali solution with the lanolin solution. The PTFE coil serves as the reactor where the saponification reaction between the alkali solution and the lanolin solution takes place. The oil bath maintains the desired reaction temperature, and the reaction temperature sensor monitors the system in real time. The product collection module utilizes a fraction collector (Model DBS-100N, Shanghai Qingpu Huxi Factory, Shanghai, China) to collect products. The environmental sensing module includes an atmospheric pressure sensor, a temperature sensor, and a humidity sensor (combined Model WY0003, Anhui Weiyu Electronic Technology Co., Ltd., Hefei, China). The SapoMind software system is executed by the central control module to monitor and control the entire hardware setup.

#### 4.2.2. Software System

**Figure 7:**
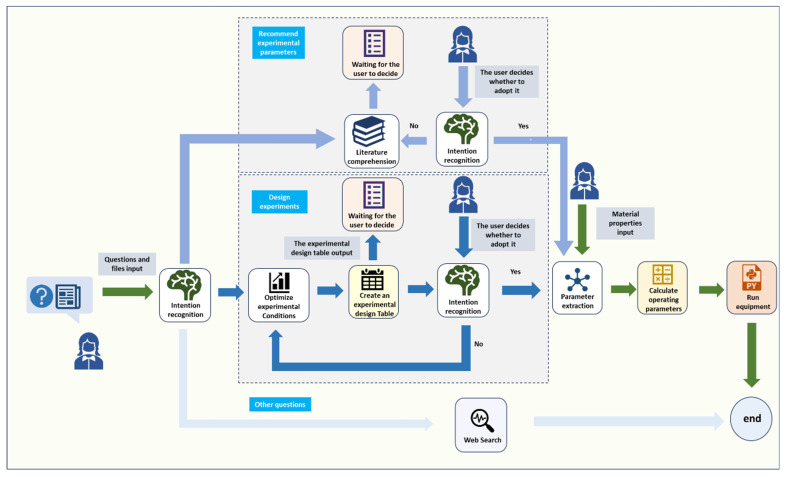
Software system workflow diagram.

The software system of SapoMind (version 1.0) was constructed using a workflow architecture as illustrated in Figure 7. It can perform process parameter recommendation, experimental design, and automated experimentation based on user requirements. Nodes filled in white indicate those that requiring LLM processing, including intent recognition, literature comprehension, parameter extraction and experimental condition optimization. The experimental design table generation node and operational-parameter calculation node are implemented through custom code. The web search node utilizes the Baidu Search API, while the hardware operation node is deployed locally and communicates with the hardware system through a Python (3.12.2) library called by SapoMind. Table 5 presents a summary of the LLM utilization status of each node. Functional descriptions of each node are as follows:

- Intent recognition node: Identifies the user’s intent and directs the subsequent workflow based on the recognition result.
- Literature comprehension node: Comprehends user-provided literature and generates experimental protocols.
- Parameter extraction node: Extracts material parameters from user input after protocol confirmation.
- Experimental condition optimization node: Qualitatively analyzes failed experiments due to improper conditions and generates optimized experimental ranges.
- Definitive Screening Design (DSD) table generation node: Generates DSD experimental tables using optimized parameters.
- Operational parameter calculation node: Converts experimental parameters, including reaction temperature, reaction time, the mass fraction of the lanolin solution, and alkali dosage, into hardware control parameters such as pump flow rate, residence time, and oil bath temperature. Pump flow rate and residence time are calculated using Equations (1)–(5):
  (1) $$Q_{1}=\frac{V_{r}}{t_{0}\times \left[1+\left(\rho_{1}\times w_{0}\times \frac{\lambda_{a}}{w_{j}\times \rho_{2}}\right)+\left(\frac{\rho_{1}\times \frac{w_{0}}{w_{i}}-\rho_{1}}{\rho_{3}}\right)\right]}$$
  (2) $${Q_{2}=(\rho }_{1}\times w_{0}\times Q_{1}\times \lambda_{a})/(w_{j}\times \rho_{2})$$
  (3) $$Q_{3}=(\rho_{1}\times w_{0}\times Q_{1}/w_{i}-\rho_{1}\times Q_{1})/\rho_{3}$$
  (4) $$t_{1}=t_{0}+t_{2}+t_{3}$$
  (5) $$t_{2}=\frac{V_{1}}{Q_{1}}$$
  where ρ_1_ represents the density of the lanolin feedstock solution; $w_{0}$ represents the mass fraction of the lanolin feedstock solution; $w_{i}$ represents the mass fraction of the lanolin solution; $w_{j}$ represents the mass fraction of the alkali solution; $\rho_{2}$ represents the density of the alkali solution; $\rho_{3}$ represents the density of the diluent; $\lambda_{a}\;$ represents the alkali dosage; $t_{0}$ represents the reaction time; $t_{2}$ represents the lanolin feedstock solution transfer time from the pump outlet to the second T-junction; $t_{3}$ represents the equilibration time (set to 2 min); $V_{1}$ represents the tubing volume between the pump and the second T-junction (set to 10 mL). $t_{1}$ represents the residence time; $Q_{1}$ represents the volumetric flow rate of the lanolin feedstock solution; $Q_{2}$ represents the volumetric flow rate of the alkali solution; $Q_{3}$ represents the volumetric flow rate of the diluent; $V_{r}$ represents the volume of the PTFE coil used for the reaction.
- Web search node: Activates Baidu search API for non-saponification-related queries.
- Hardware operation node: Executes pre-generated Python communication scripts for hardware control.
- User interaction node: Outputs experimental protocols and awaits user confirmation before proceeding.

#### 4.2.3. Optimization Strategies for LLMs

Preliminary Screening of LLMs: The intent recognition node and parameter extraction node employ ERNIE 4.5-Turbo-32K. For the literature comprehension node, experimental condition optimization node, and hardware operation node (including pre-generation of control codes for each device), the selected LLMs were obtained through screening and optimization.

The performance of 10 LLMs across three distinct tasks was compared. The evaluated models include ERNIE X1-Turbo-32K, ERNIE 4.5-Turbo-32K, DeepSeek-R1, DeepSeek-V3, QwQ-32B, QwQ2.5-Max, Hunyuan-Large, Meta-Llama-3-8B, Llama-2-7B-Chat, and ChatGLM2-6B-32K. The three tasks are defined as follows: (1) Literature comprehension: The models were required to extract key conditions of saponification processes from studies and present the findings in Markdown format (rendered as tables in HTML page); (2) Code generation: The models were tasked with generating Python control code for the hardware devices based on the communication protocols; (3) Experimental condition optimization: Based on the input process conditions and results, the models predicted the range of conditions that meet user requirements. The predicted range was output in JSON format, while the reasoning was provided in TXT format. Detailed descriptions are provided in Section S2 of Supplementary Material File S1.

Dataset Collection: The dataset construction was divided into two stages: literature extraction and model-generated sample supplementation. First, 100 relevant studies on process optimization meeting the above research criteria were retrieved and collected. Through batch inference with an LLM, we extracted the process optimization objectives, pre-optimization experimental conditions and results from the literature as prompts, the parameter ranges of optimization experiments as responses, and the basis for selecting parameter ranges as reasoning content. This yielded a 100-sample literature-based dataset, which was then manually verified. Based on this basic dataset, an LLM was used to generate supplementary samples; after manual screening, 102 qualified samples were obtained, and the samples were finally integrated into 202 samples for SFT. The full dataset is provided in Supplementary Material File S2.

This dataset covers the typical process range of lanolin saponification, with the distribution of training data for each key parameter as follows: 120 temperature-related samples (with a high-frequency range of 30–90 °C), 122 reaction time-related samples (with a high-frequency range of 0.5–120 min), 56 concentration-related samples (with a high-frequency range of 6% (*w*/*w*)–50% (*w*/*w*)), and 52 reactant ratio-related samples (with a high-frequency range of 0.6–5). In addition, it includes 21 samples of process conversion from batch production to microchannel continuous-flow production, and all data match the actual process requirements of lanolin saponification.

Fine-tuning of LLMs: The LLM was fine-tuned using supervised fine-tuning (SFT) and reinforcement fine-tuning (RFT). In the SFT, the full-parameter fine-tuning training method was employed, with the following parameter settings: 10 training epochs, a learning rate of 0.000001, and a global batch size of 16. In the RFT, the full-parameter fine-tuning training was applied, guided by a reward rule based on string similarity comparison. The reinforcement learning method used was Group Relative Policy Optimization (GRPO). The training was conducted for 5 epochs, with 8 samples generated per prompt. A 10-case verification set was built using the better-performing fine-tuned model to validate its optimization capability.

### 4.3. SapoMind Hardware–Software Integration

SapoMind comprises two core components: intelligent agent interaction and hardware system status display. To visually present both components concurrently, a local web page deployed on the central control module was developed.

Among them, the intelligent agent interaction module was deployed via the Baidu AI Cloud platform (https://cloud.baidu.com) in the form of predefined workflows and embedded into the local web page through iframe embedding. The relevant technical details are as follows:

API Endpoint: https://wx.baeapps.com/api/ai_apaas/v1/web_embed/conversation. accessed via iframe embedding and SDK authorization (accessed on 21 March 2025).

The request payload parameters for DeepSeek-R1-Distill-Qwen-32B-Data Analysis are set as follows: temperature = 0.6; top_p = 0.95; penalty_score = 1; max_tokens = 2048. For ERNIE 4.5-Turbo-32K and DeepSeek-R1, the request payload parameters adopt the default values of the models.

Response Format: JSON.

As illustrated in Supplementary Material File S1, Figure S15, the local interactive interface supports multiple functionalities, including user interaction with SapoMind, real-time visualization of sensor data, and logging of hardware system operation records.

### 4.4. Analytical Method

The cholesterol and lanosterol content in the lanolin saponification solution was analyzed using high-performance liquid chromatography (HPLC; Vanquish, Thermo Fisher Scientific, Waltham, MA, USA) equipped with a variable wavelength detector (VWD). The chromatographic conditions were adapted from the multi-analyte quantification method established by Kaidierya et al. [39], and detailed procedures and representative chromatograms are provided in Section S4 of Supplementary Material File S1.

### 4.5. Data Processing

#### 4.5.1. Process Performance Indicators

The product quality, process efficiency, and environmental impact were evaluated using the following indicators: the yields of cholesterol and lanosterol during the saponification process, the carbon emissions, the alkali dosage and the residence time. The calculation methods for the yields of cholesterol and lanosterol are provided in Section S5 of Supplementary Material File S1.

The calculation formula for carbon emissions per unit of processed lanolin was derived from the “Guidelines for Accounting Methods and Reporting of Greenhouse Gas Emissions from Chemical Production Enterprises in China” [26], as shown in Equations (6)–(8).

(6) $${F_{1}=Q}_{1}\times \rho_{1}\times w_{0}$$

(7) $${F_{2}=Q}_{2}\times \rho_{2}\times w_{j}$$

(8) $$m_{CO_{2}}=\frac{\sum_{i=1}^{n}\left(F_{i}\times c_{i}\right)\times (T-25)\times C\times \eta \times M_{{co}_{2}}}{LHv\times M_{c}\times F_{1}\times 10^{6}}$$

where $F_{i}$ represents the mass flow rate, kg/min; $c_{i}$ represents the specific heat capacity, J/(kg × °C); *n* represents the number of substance types in the system, where the subscript *i* represents individual substances, 1 represents lanolin, and 2 represents alkali; *T* represents reaction temperature, °C; *C* represents the carbon content per unit volume of fuel with a value of 15.3, t C/(10^4^ × Nm^3^); *η* represents the carbon oxidation rate of the fossil fuel with a value of 0.99; $M_{{CO}_{2}}$ represents the molar mass of CO_2_ with a value of 44 g/mol; $M_{c}$ represents the molar mass of carbon with a value of 12 g/mol; and LHv represents the lower heating value of natural gas with a value of 389.9, GJ/(10^4^ × Nm^3^).

#### 4.5.2. Data Modeling

A quantitative model between lanosterol yield and process conditions was established using Equation (9). The model was simplified through backward stepwise regression in Design Expert 12.0.1.0 (Stat-Ease Inc., Minneapolis, MN, USA), with the significance level set at 0.10.

(9) $$Y_{LANOSTEROL}=a_{0}+\sum_{i=1}^{4}a_{i}X_{i}+\sum_{i=1}^{4}a_{ii}X_{i}^{2}+\sum_{i=1}^{3}\sum_{j=i+1}^{4}a_{ij}X_{i}X_{j}$$

where $a_{0}$ represents the constant term; $a_{i}$, $a_{ii}$, and $a_{ij}$ represent the regression coefficients for linear, quadratic, and interaction terms, respectively; $X_{i}$ and $X_{j}$ represent the process conditions; and $Y_{LANOSTEROL}$ represents the lanosterol yield.

To ensure the robustness of the optimized process parameters, a risk quantification method based on Monte Carlo simulation was employed to select robust conditions [40,41,42]. The significance level was set at 0.050, the acceptable risk was 0.2, and the number of simulations was 500.

#### 4.5.3. Sensitivity Analysis of Carbon Emissions

The procedure for analyzing the global sensitivity of carbon emissions to each process parameter is as follows [43,44]:

Monte Carlo Simulation: Carbon emissions were evaluated via 10,000 stochastic simulations. During the simulations, process parameters were generated within their respective ranges using simple random sampling: reaction temperature of 70–90 °C, reaction time of 5–15 min, mass fraction of lanolin solution of 0.15–0.35, and alkali consumption of 0.115–0.125 kg/kg lanolin.

Correlation and Significance Analysis: Pearson correlation coefficients between each process parameter and carbon emissions were calculated, and the significance *p*-values were derived via the *t*-test. Significance levels were classified according to the criteria: *p* < 0.05 (significant) and *p* > 0.05 (non-significant).

Screening of Key Parameters: Based on the absolute values of the correlation coefficients, all process parameters were categorized into three grades: extremely sensitive (|r| > 0.5), weakly sensitive (0.2 < |r| < 0.5), and insensitive (|r| < 0.2). This classification allowed for the identification of the core influencing factors of carbon emissions.

## 5. Conclusions

An intelligent agent called SapoMind was successfully developed by integrating LLMs with a continuous-flow microreactor. SapoMind was then used for the optimization of lanolin saponification. By leveraging microchannels to enhance mass and heat transfer, the system significantly improved the atom economy and process controllability of the saponification reaction.

After comparison and optimization of LLMs, DeepSeek-R1 was employed for literature comprehension and code generation, while the supervised fine-tuned DeepSeek-R1-Distill-Qwen-32B was used for experimental condition optimization. In addition, ERNIE 4.5-Turbo-32K was applied for intention recognition and parameter extraction.

SapoMind autonomously generated and executed 15 experimental designs within 256 min. The mathematical model derived from the experimental results achieved a coefficient of determination (R^2^) exceeding 0.9, accurately quantifying the relationships among saponification conditions and lanosterol yield. The risk quantification method combining the exhaustive method with Monte Carlo simulation was employed to identify robust and green experimental conditions. The final optimized process conditions are as follows: a reaction temperature of 70 °C, a reaction time of 5 min, a lanolin solution mass fraction of 25%, and an alkali dosage of 116 mg/g of lanolin. Under these optimized conditions, cholesterol yield reached 100%, while lanosterol yield reached approximately 65%. The resulting lanolin alcohol product complied with European Pharmacopoeia standards for cholesterol and lanosterol content.

Compared to conventional batch processes, the system reduced carbon emissions by 53%, solid waste by 37%, and shortened reaction time in the reactor to merely 540 s. Overall, the optimized saponification process aligns with green chemistry principles. The agent-driven optimization approach demonstrates advantages in labor savings and operational efficiency.

## Figures and Tables

**Table 1:** Analysis of variance.

Term	F Value	*p* Value
Reaction temperature (°C)	19.09	0.0024
Reaction time (min)	15.69	0.0042
Lanolin solution mass fraction	29.17	0.0006
Reaction temperature (°C) × Lanolin solution mass fraction	10.24	0.0126
Model	18.55	0.0004

**Table 2:** Significance and sensitivity analysis.

Name	Correlation Coefficient	*p* Value	Significance Level	Sensitive Level
Alkali consumption (kg/kg lanolin)	0.0059	0.553	Not significant	Insensitive
Reaction time (min)	0.0068	0.495	Not significant	Insensitive
Reaction temperature (°C)	0.4497	0.000	Significant	Sensitive
Lanolin solution mass fraction	−0.8673	0.000	Significant	Extremely sensitive

**Table 3:** Optimized process conditions and results.

Name	Value
Reaction temperature (°C)	70.0
Reaction time (min)	5.0
Lanolin solution mass fraction	0.25
Alkali consumption (kg/kg lanolin)	0.116

**Table 4:** The results of the literature comparison.

Reaction Time	Alkali Consumption (kg/kg Lanolin)	Reaction Temperature (°C)	Lanolin Solution Mass Fraction	Reference
40 min	0.110	160	25%	[4]
4 h	0.114	80	33%	[32]
1.5 h	0.360	60	16%	[33]
9 min	0.116	70	25%	This study

**Table 5:** Summary of SapoMind Nodes and Their Driving Modes.

Node Name	Whether LLM-Driven
Intent recognition	Yes
Literature comprehension	Yes
Parameter extraction	Yes
Experimental condition optimization	Yes
Definitive Screening Design (DSD) table generation	No
Operational parameter calculation	No
Web search	No
Hardware operation	No
User interaction	No

## Data Availability

The original contributions presented in this study are included in the article and Supplementary Materials. Further inquiries can be directed to the corresponding author.

## Supplementary Materials

The following supporting information can be downloaded at: https://www.mdpi.com/article/10.3390/ph19020264/s1, Figure S1: Partial output of the literature comprehension task for Qwen2.5-Max before prompt optimization; Figure S2: Partial output of the literature comprehension task for Qwen2.5-Max after prompt optimization; Figure S3: Partial output of the code generation task for QwQ-32B before prompt optimization; Figure S4: Partial output of the code generation task for QwQ-32B after prompt optimization; Figure S5: Input example 1 for the experimental condition optimization task; Figure S6: The output of the experimental condition optimization task for DeepSeek-R1-Distill-Qwen-32B before SFT in example 1; Figure S7: The output of the experimental condition optimization task for DeepSeek-R1-Distill-Qwen-32B after SFT in example 1; Figure S8: Input example 2 for the experimental condition optimization task; Figure S9: The output of the experimental condition optimization task for DeepSeek-R1-Distill-Qwen-32B before SFT in example 2; Figure S10: The output of the experimental condition optimization task for DeepSeek-R1-Distill-Qwen-32B after SFT in example 2; Figure S11: The training/validation loss curve and validation token accuracy of DeepSeek-R1-Distill-Qwen-32B using SFT; Figure S12: Detailed interaction of the question-answering process during automated execution under literature-based conditions; Figure S13: Detailed interaction of the question-answering process automated experimental design and execution(1); Figure S14: Detailed interaction of the question-answering process automated experimental design and execution(2); Figure S15: The interactive interface of SapoMind; Figure S16. HPLC of saponification solution (1: cholesterol; 2: lanosterol). Figure S17 The relationship between the lanosterol/cholesterol peak area ratio and the lanosterol/cholesterol yield. Table S1: Pre-optimization prompt; Table S2: Scoring criteria; Table S3: Scores for different indicators; Table S4: Optimized prompt; Table S5: Contributions of greenness score items; Section S1: Results of preliminary screening and prompt optimization for LLMs; Section S2: Method of preliminary screening and prompt optimization for LLMs; Section S3: Pre-operation hardware system preparation; Section S4: Analytical methods for lanolin saponification solution; Section S5: Calculation of cholesterol and lanosterol yield.
