# Supplementary material for: An LLM-Based Intelligent Agent and Its Application in Making the Lanolin Saponification Process Greener
Source: Pharmaceuticals (Basel). 2026 Feb 3;19(2):264. doi: 10.3390/ph19020264 (PMC12943705; doi:10.3390/ph19020264)
Supplement: Supplementary file 1 [file pharmaceuticals-19-00264-s001.zip › Supplementary files/Supplementary Material File S1.pdf]

Supplementary Material File S1

An LLM-Based Intelligent Agent and Its Application in Making the Lanolin Saponification Process Greener

Qinglin Wang [a, b], Yu Wang [a, b], Xingchu Gong [a, b, c]

a. Pharmaceutical Informatics Institute, College of Pharmaceutical Sciences, Zhejiang University, Hangzhou, 310058, China

b. Jinhua Institute of Zhejiang University, Jinhua, 321016, China

c. National Key Laboratory of Chinese Medicine Modernization, Zhejiang University, Hangzhou, 310058, China

*Correspondence: gongxingchu@zju.edu.cn; Tel.: +86-571-88208426

S1 Results of preliminary screening and prompt optimization for LLMs

The accuracy of ten commonly used LLMs was compared across three tasks. In the literature comprehension task, the primary error observed across models was the inclusion of non-saponification process conditions and parameter ranges in their generated outputs. For code generation, common errors involved: (1) incorrect register address identification, (2) unit conversion errors between input data and preset values for pumps and oil baths, and (3) overly verbose code due to failure to import external libraries. All models demonstrated suboptimal performance in the experimental condition optimization task. Among all evaluated models, DeepSeek-R1, DeepSeek-V3, QwQ-32B and QwQ2.5-Max consistently achieved superior performance, particularly excelling in literature comprehension (evidenced by higher scores). Consequently, these models were selected for subsequent optimization procedures.

Based on a preliminary analysis of the LLMs' outputs, prompt structures were refined through four key strategies: (1) clarifying task objectives, (2) explicitly defining output formats, (3) incorporating representative examples, and (4) emphasizing the logical relationships between experimental conditions and outcomes.

The refined prompts notably enhanced performance in both literature comprehension and code generation tasks. Subsequent validation experiments, comprising 10 trials per task, yielded consistently high accuracy, thereby confirming the robustness and reliability of the optimized prompt structures. Among the tested models, DeepSeek-R1 outperformed QwQ-32B in the validation phase. Therefore, DeepSeek-R1 was employed in the literature comprehension node and for the pre-generation of control code in the hardware execution node of SapoMind.

S2 Method of preliminary screening and prompt optimization for LLMs

The initial prompt templates used for LLMs selection are shown in Supplementary Material 1, Table S1. Each task was scored on a 5-point scale according to the criteria in Supplementary Material 1, Table S2. In addition to the 5-point subjective scoring method, two objective metrics — BLEU and ROUGE (including ROUGE-1 and ROUGE-2, with a full score of 100 for each)— were adopted for comparative analysis. The results indicate that BLEU and ROUGE exhibit a positive correlation with subjective scores only in partial scenarios, subjective scoring can better focus on the core information of the task, being more intuitive and accurate. Additionally, Ruan, Y et al. (Nat Commun 15, 10160 (2024)) and Boiko, D.A et al. (Nature 624, 570 (2023)) both adopted subjective scoring as the evaluation method during the LLM screening stage for agent construction, which also demonstrates the feasibility of this approach. and the detailed evaluation results are presented in Table S3.

For each task, three different files were selected. The score of the LLM on each task was calculated as the average score obtained across all instances of that task.

Prompt Optimization: After initially selecting the LLMs with relatively higher scores, we optimized the prompts based on the models' output results to enhance task execution accuracy. The refined prompts are detailed in Supplementary Material 1, Table S4. The highest-scoring LLM was selected as the foundation for constructing the SapoMind. To validate the performance of the optimized prompts, we compiled a verification set consisting of 10 saponification research papers and communication protocols. This verification set was used to evaluate the optimized LLM's performance in Task 1 and Task 2. The literature comprehension node and the pre-generation of control codes for devices in the hardware operation node were implemented using the LLMs with the highest score.

Table S1. Pre-optimization prompt*

Task name	Prompt
Literature comprehension	Identify the critical conditions for saponification based on literature and summarize their corresponding ranges in Markdown format.
Code generation	Generate Python code for device communication according to the uploaded communication protocol.
Experimental condition optimization	Predict the range of conditions that meet user's requirements based on the process conditions and results. The parameter ranges for optimization experiments should be provided in JSON format, along with justification in TXT format.

*In the experiments, all the prompts were in Chinese. To facilitate a better understanding for readers, the prompts are translated into English here.

Table S2. Scoring criteria

Task title	Task detail	Task score	Remark
Literature Comprehension	The critical conditions for saponification were correctly identified/established.	2	
	The critical process parameter ranges for saponification were validated as correct.	1	
	The summary was presented in Markdown	1	

	format.		
	No information was provided on processes other than saponification.	1	
Code Generation	The code was correct.	2	If the code contained errors, this item deserved a score of 0.
	The code was concise.	1	
	The code was robust.	1	
	The code was readable.	1	
Experimental condition optimization	The process parameter ranges for the optimization experiments were validated as correct.	3	
	The justification for the recommendation was scientifically valid.	1	
	The output format was correct.	1	

Table S3. Scores for different indicators

Model name	Literature comprehension				Code generation				Experimental condition optimization			
	5-point scoring	Rouge-1	Rouge-2	BLEU	5-point scoring	Rouge-1	Rouge-2	BLEU	5-point scoring	Rouge-1	Rouge-2	BLEU
ERNIE X-Turbo-32K	3.67	18.56	5.04	1.75	3.00	14.58	5.88	3.28	1.00	6.75	1.40	1.49
ERNIE 4.5-Turbo-32K	3.67	12.66	3.02	2.73	4.00	19.81	7.79	5.59	0.67	14.81	6.67	4.18
DeepSeek-R1	4.67	44.64	32.05	23.86	4.67	19.24	4.48	2.75	2.33	11.11	5.13	5.45
DeepSeek-V3	4.00	35.23	17.31	14.47	4.00	20.35	7.52	4.38	2.00	15.82	4.57	5.84
QwQ-32B	4.67	41.27	11.56	11.02	4.00	20.22	7.23	3.83	1.33	18.75	5.71	6.71
QwQ2.5-Max	4.00	29.04	6.60	3.73	4.00	18.40	6.30	4.01	1.00	13.95	4.26	4.67
Hunyuan-Large	3.67	23.98	7.72	5.55	3.67	16.33	8.28	2.18	0.67	6.14	2.10	2.44
Meta-Llama-3-8B	4.00	30.30	15.56	8.29	4.00	17.42	5.8	3.67	0.67	10.21	4.77	4.54
Llama-2-7B-Chat	4.00	22.34	7.26	3.66	4.00	18.27	7.28	2.41	0.67	6.20	1.89	1.77
ChatGLM2-6B-32K	3.67	11.38	2.76	2.54	4.00	20.75	6.82	3.85	0.67	4.8	1.08	1.34

Table S4. Optimized prompt*

Task name	Prompt
Literature Comprehension	You are a literature analysis expert skilled in reviewing and summarizing research papers.<br>1. Carefully analyze the literature to understand the experimental methods and results related to saponification.<br>Note: Focus only on the saponification process itself. Do not include any post-saponification steps!<br>2. Based on the saponification conditions and results, recommend the critical process parameters and their optimal ranges.<br>3. Ensure the response is clear, concise, and supported by sufficient evidence. Present the findings in a Markdown format. Let's think step by step.
Code Generation	You are an automation control expert skilled in generating Python communication code based on communication protocols.<br>1. Use Python external libraries to ensure the code is concise and clear, strictly following the command format, data exchange method, and communication parameters specified in the protocol.<br>2. Carefully read the communication protocol, paying special attention to correct register addressing to ensure accurate device control.<br>3. Carefully review the provided examples, noting the conversion of read/write data with decimal points.
Experimental condition optimization	You are an expert in experimental optimization, specializing in analyzing experimental data and predicting the range of experimental conditions that are likely to meet the user's specified requirements.<br>1. A detailed analysis is conducted to evaluate the impact of each experimental condition on the outcome.<br>2. The optimized experimental conditions are provided in JSON format, specifying value ranges rather than single-point values.<br>3. The reasoning behind the recommendations is documented in a separate TXT file.

*In the experiments, all the prompts were in Chinese. To facilitate a better understanding for readers, the prompts are translated into English here.

Table S5 Contributions of greenness score items

NO.	Name	Before optimization	After optimization
I	Prevention waste	5	5
II	Atom Economy	5	8
III	Less Hazardous Chemical Syntheses	8	8
IV	Designing Safer Chemicals	10	10
V	Safer Solvents and Auxiliaries	8	8
VI	Design for Energy Efficiency	5	8
VII	Use of Renewable Feedstocks	8	8
VIII	Reduce Derivatives	8	8
IX	Catalysis	5	5
X	Design for Degradation	10	10
XI	Real-time analysis for Pollution Prevention	5	5
XII	Inherently Safer Chemistry for Accident Prevention	5	10
	Total	82	93

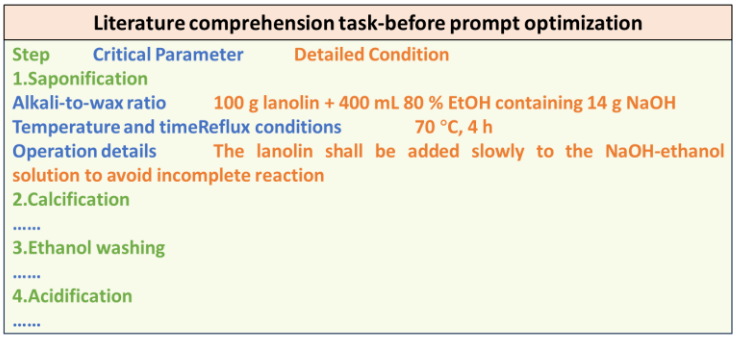

Fig. S1. Partial output of the literature comprehension task for Qwen2.5-Max before prompt optimization. Two errors were identified: (1) inclusion of process conditions irrelevant to saponification; (2) the output was not in Markdown format.

*In the experiment, the output was in Chinese. To facilitate a better understanding for readers, the output is translated into English here.

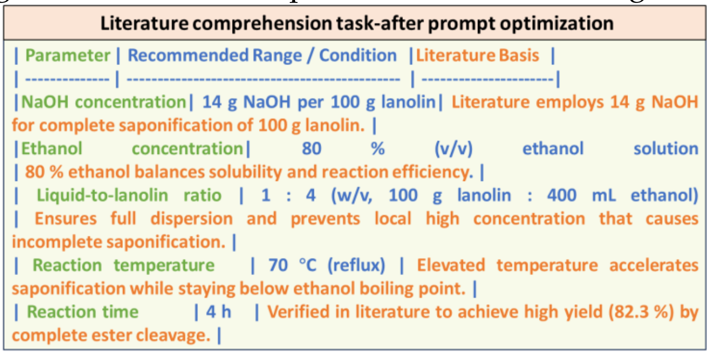

Fig. S2. Partial output of the literature comprehension task for Qwen2.5-Max

after prompt optimization. The conditions were presented in Markdown format (rendered as tables in HTML page), and no non-saponification processes were included.

*In the experiment, the output was in Chinese. To facilitate a better understanding for readers, the output is translated into English here.

Code generation task-before prompt optimization

```
def set_flow_rate(self, flow_rate: int):
    """Set pump flow rate (unit: refer to documentation)"""
    if not 0 <= flow_rate <= 65535:
        raise ValueError("Flow rate must be between 0 and 65535.")
    response = self.client.write_register(
        address=0x0006,  # Pump flow-rate register address
        value=flow_rate,
        unit=self.address,
    )
    self._check_response(response)
    print(f"Flow rate set to {flow_rate}")

def start_pump(self):
    """Start pump (function code 0x05, coil address 0x0001)"""
......
```

Fig. S3. Partial output of the code generation task for QwQ-32B before prompt optimization. A register address error was identified in the generated control code.

*In the experiment, the output was in Chinese. To facilitate a better understanding for readers, the output is translated into English here.

Code generation task-after prompt optimization

```
def set_min_pressure(self, pressure):
    """Set minimum pressure"""
    return self._write_register(3, int(pressure))

def set_flow_rate(self, flow_ml_per_min):
    """Set flow rate (unit: mL/min; internally converted to 0.001 mL/min units)"""
    value = int(flow_ml_per_min * 1000)  # Convert to 0.001 mL/min units
    return self._write_register(5, value)

def start_pump(self):
    """Start pump"""
    return self._write_coil(0x0001, True)
......
```

Fig. S4. Partial output of the code generation task for QwQ-32B after prompt optimization. The register address error was corrected.

*In the experiment, the output was in Chinese. To facilitate a better understanding for readers, the output is translated into English here.

Experimental condition optimization task : Input example 1

You are an expert in experimental optimization, specializing in analyzing experimental data and predicting the range of experimental conditions that are likely to meet the user's specified requirements.
1. A detailed analysis is conducted to evaluate the impact of each experimental condition on the outcome.
2. The optimized experimental conditions are provided in JSON format, specifying value ranges rather than single-point values.
3. The reasoning behind the recommendations is documented in a separate TXT file.

Here are the collected experimental conditions and results. Please help me optimize the experimental conditions. My requirements are as follows::
Requirements: The extract yield should be no less than 8%, with higher process efficiency and greenness preferred.

Experimental Conditions and Results:
{"No.": [1, 2, 3, 4, 5, 6, 7, 8, 9, 10, 11, 12, 13, 14],
"Extraction Times": [1, 2, 3, 4, 5, 2, 2, 2, 2, 2, 2, 2, 2, 2],
"Extraction Time (h)": [0.5, 0.5, 0.5, 0.5, 0.5, 0.5, 1.0, 1.5, 2.0, 2.5, 0.5, 0.5, 0.5, 0.5],
"Solid-to-Liquid Ratio": [0.1, 0.1, 0.1, 0.1, 0.1, 0.1, 0.1, 0.1, 0.1, 0.1, 0.125, 0.1, 0.0833, 0.0714],
"Extract Yield (%)": [4.0, 9.0, 10.0, 10.5, 11.0, 9.0, 9.5, 10.0, 10.5, 9.5, 8.5, 9.0, 11.0, 11.5]}

Fig. S5. Input example 1 for the experimental condition optimization task
*In the experiment, the input was in Chinese. To facilitate a better understanding for readers, the input is translated into English here.

Experimental condition optimization task-Output example 1 before SFT

{
"extraction_cycles": [2],
"extraction_time_h": [1.0, 2.0],
"solid_to_liquid_ratio": [0.0833, 0.1]
}
Rationale (TXT format)
Based on the experimental data and the targets (extract yield ≥ 8 %, highest possible process efficiency and greenness), the following recommendations are made:
Extraction cycles
– Recommended range: 2 cycles
– Rationale: Two extraction cycles give a high extract yield (9.0 %–11.53 %) with good process efficiency and greenness. More cycles (e.g., 5) markedly lower the yield.
Extraction time
– Recommended range: 1.0–2.0 h
– Rationale: 1.0 h already affords 10.5 % yield, and 2.0 h maintains the same level. Shorter times (e.g., 0.5 h) are insufficient, while longer times offer no gain.
Solid-to-liquid ratio
– Recommended range: 0.0833–0.1
– Rationale: A ratio of 0.1 delivers the highest yield (11.53 %); 0.0833 is the lower bound that still meets the ≥ 8 % target.

Fig. S6. The output of the experimental condition optimization task for DeepSeek-R1-Distill-Qwen-32B before SFT in example 1. Error point: The extraction time was too long and the solid-to-liquid ratio was too low. Although the extract yield requirement was met, the optimization did not consider the requirements for process efficiency and greenness.
*In the experiment, the output was in Chinese. To facilitate a better understanding for readers, the output is translated into English here.

Experimental condition optimization task-Output example 1 after SFT

```
{
  "extraction_cycles": [1, 2],
  "extraction_time_h": [0.3, 0.5],
  "solid_to_liquid_ratio": [0.13, 0.17]
}
```

Recommended rationale (TXT format)

Extraction cycles

Experimental data show that 2 cycles already deliver an extract yield ≥ 9.0 %, meeting the ≥ 8 % requirement. Further increases (3 cycles or more) raise the yield only marginally while increasing resource consumption and time, lowering greenness and efficiency. Therefore, keep cycles between 1 and 2 to save resources and improve sustainability.

Extraction time (h)

Keeping the time in the 0.3–0.5 h window gives yields of 8.5 %–9.0 %, again satisfying the ≥ 8 % target. Longer times (≥ 1.0 h) improve yield only slightly but raise energy use and processing time, reducing greenness and efficiency. Hence, 0.3–0.5 h is recommended.

Solid-to-liquid ratio

Ratios between 0.13 and 0.17 provide yields of 8.5 %–10.0 %, well above the 8 % threshold. Ratios below this range (e.g., 0.0625) do not offer further advantages.

Fig. S7. The output of the experimental condition optimization task for DeepSeek-R1-Distill-Qwen-32B after SFT in example 1. The response takes into account the balance among greenness, process efficiency, and extract yield.

*In the experiment, the output was in Chinese. To facilitate a better understanding for readers, the output is translated into English here.

Experimental condition optimization task : Input example 2

You are an expert in experimental optimization, specializing in analyzing experimental data and predicting the range of experimental conditions that are likely to meet the user's specified requirements.

1. A detailed analysis is conducted to evaluate the impact of each experimental condition on the outcome.
2. The optimized experimental conditions are provided in JSON format, specifying value ranges rather than single-point values.
3. The reasoning behind the recommendations is documented in a separate TXT file.

Here are the collected experimental conditions and results of a batch reaction experiment for p-aminobenzoic acid synthesis in a stirred tank reactor. Please help optimize the experimental conditions with the following requirements:

Requirements:

1.Replace the reaction setup with a microchannel continuous-flow reactor .

2.Maintain a conversion rate greater than 99% and a selectivity greater than 82%

Experimental Conditions and Results:

```
{"Original Batch Reaction Conditions": {
   "Reaction Temperature": "150–160 °C",
   "Reaction Time": "8 hours",
   "Reagents": "p-Nitrotoluene-4-sulfonic acid (p-NTS), potassium hydroxide (KOH), water (H₂O)",
   "Molar Ratio (p-NTS:KOH:H₂O)": "1:3.3:17"
 },
 "Results": {
   "Conversion Rate": "99%",
   "Selectivity": "82%" }
}
```

Fig. S8. Input example 2 for the experimental condition optimization task

*In the experiment, the input was in Chinese. To facilitate a better understanding for readers, the output is translated into English here.

Experimental condition optimization task -Output example 2 before SFT

```
{
 "reaction_temperature": "120–140 °C",
 "reaction_time": "1–2 h",
 "molar_ratio_p-NTS_KOH_H2O": "1 : 2.5 : 12",
}
```

Recommended rationale (TXT format)
Reaction temperature
Micro-channel reactors provide excellent heat-transfer efficiency, allowing the same conversion as batch reactors at lower temperatures. Keeping the temperature at 120–140 °C suppresses side reactions and improves selectivity.
Reaction time
The high mass-transfer performance of micro-channel reactors lets the reaction finish in a shorter time. Shortening the residence time to 1–2 h markedly increases throughput without sacrificing conversion.
Molar ratio
A feed ratio of 1 : 2.5 : 12 (p-NTS : KOH : H_2O) is close to the stoichiometric requirement. This maximizes reactant utilization, minimizes by-product formation and raises product purity.

Fig. S9. The output of the experimental condition optimization task for DeepSeek-R1-Distill-Qwen-32B before SFT in example 2. Error point: The predicted reaction time was overly long due to insufficient understanding of the microchannel reactor's characteristics.
*In the experiment, the output was in Chinese. To facilitate a better understanding for readers, the output is translated into English here.

Experimental condition optimization task-Output example 2 after SFT

```
{
 "reaction_temperature": "130–150 °C",
 "reaction_time": "5–15 min",
 "molar_ratio_p-NTS_KOH_H2O": "1 : 4 : 17"
}
```

Recommended rationale (TXT format)
Reaction temperature
Raising the temperature to 130–150 °C exploits the superior heat-transfer capability of the micro-channel continuous-flow reactor. The enhanced thermal profile accelerates the reaction while maintaining high selectivity.
Reaction time
Shortening the residence time to 5–15 min significantly boosts productivity. The combined high heat- and mass-transfer rates in the micro-channel device allow the transformation to reach completion within this narrow window.
Molar ratio
Maintaining a feed ratio of 1 : 4 : 17 (p-NTS : KOH : H_2O) ensures full conversion. The excess potassium hydroxide increases both conversion and selectivity by driving the equilibrium and suppressing side reactions.

Fig. S10. The output of the experimental condition optimization task for DeepSeek-R1-Distill-Qwen-32B after SFT in example 2. The reaction time predicted is closer to the values reported in the literature.
*In the experiment, the output was in Chinese. To facilitate a better understanding for readers, the output is translated into English here.

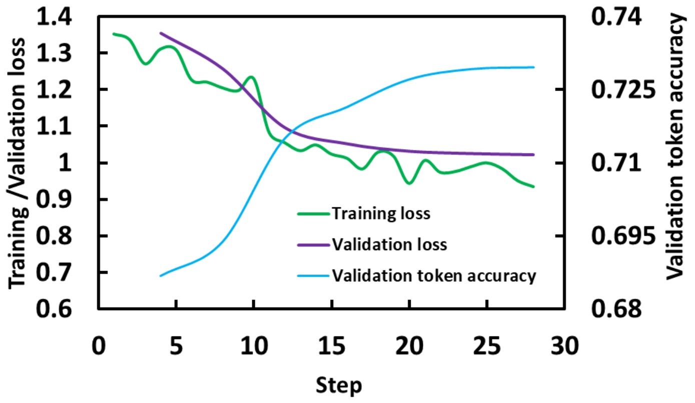

Fig. S11. The training/validation loss curve and validation token accuracy of DeepSeek-R1-Distill-Qwen-32B using SFT.

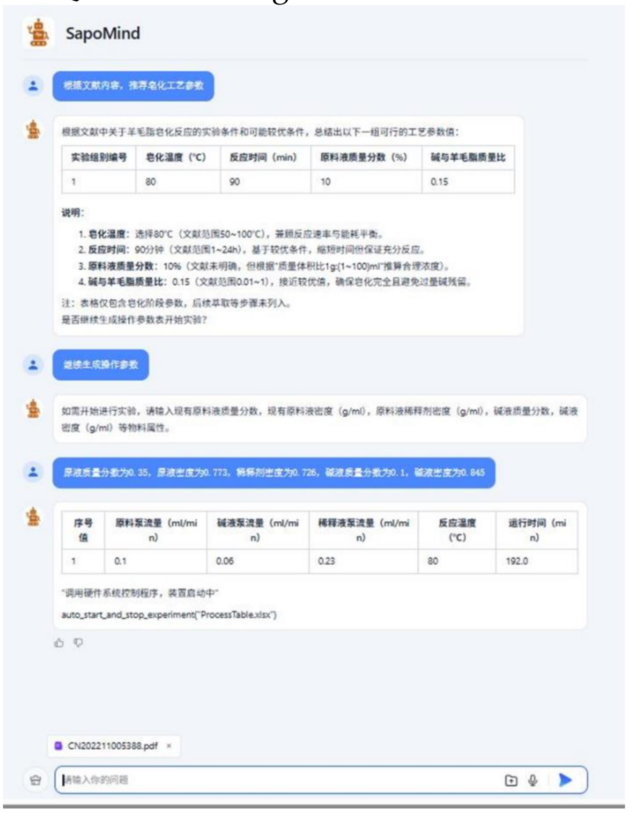

Fig. S12. Detailed interaction of the question-answering process during automated execution under literature-based conditions.

*The output is translated into English in the manuscript Figure2(a).

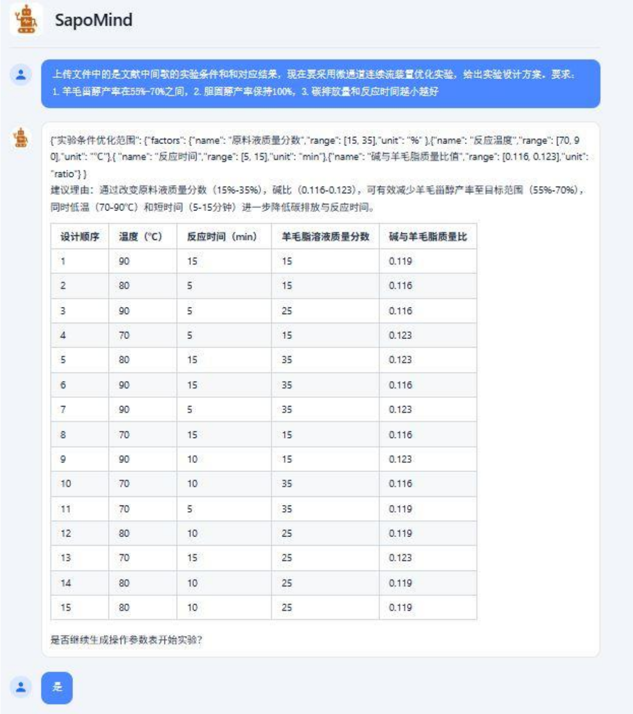

Fig. S13. Detailed interaction of the question-answering process automated

experimental design and execution(1)
*The output is translated into English in the manuscript Figure3(a).

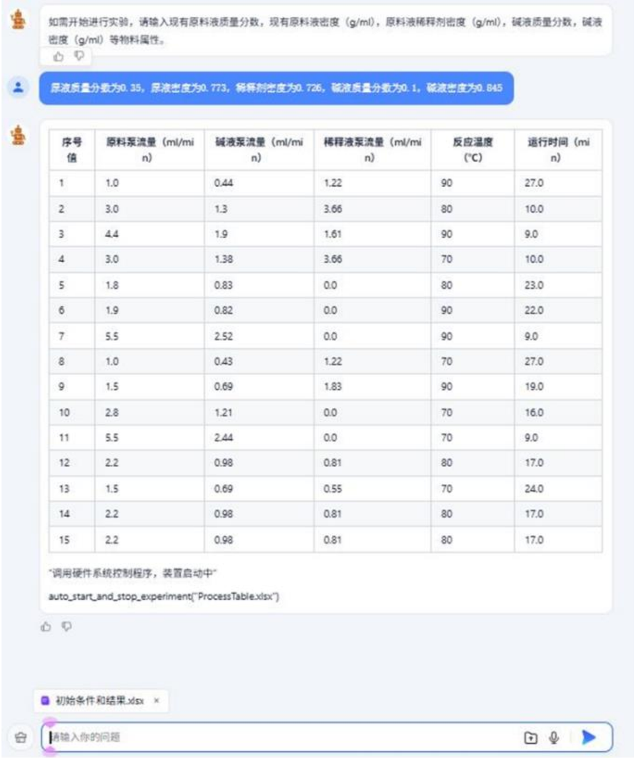

Fig. S14. Detailed interaction of the question-answering process automated experimental design and execution(2)
*The output is translated into English in the manuscript Figure3(a).

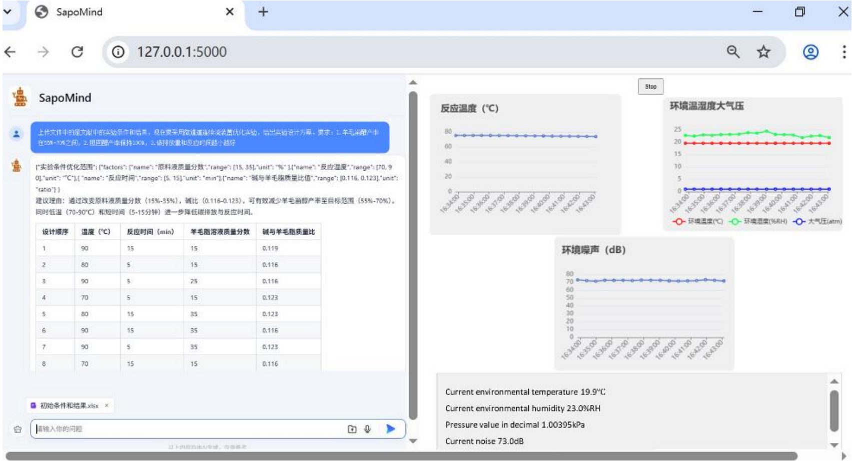

Fig. S15. The interactive interface of SapoMind

S3 Pre-operation hardware system preparation

The master switches of all hardware system modules were turned on, and the saponification reaction module was preheated. The lanolin feedstock solution was placed into the water bath for temperature maintenance in the fluid storage module. The lanolin feedstock solution, diluent, and alkali solution were loaded into their respective pump pipelines.

Diluent preparation: A 20% (w/w) n-butanol–n-octane mixed solvent was

prepared by adding n-butanol to n-octane and mixing under continuous stirring.

Lanolin feedstock solution preparation: A 35% (w/w) lanolin feedstock solution was prepared by dissolving a specified amount of lanolin in the diluent with heating.

Alkali solution preparation: A 10% (w/w) potassium hydroxide solution was prepared by dissolving a specified amount of potassium hydroxide in n-butanol with heating.

S4 Analytical methods for lanolin saponification solution

The analysis was performed using a SunShell C18 column (4.6 mm ×150 mm, 2.6 μm) with an acetonitrile (B)-water (A) mobile phase system. The flow rate was set at 1.58 mL/min with detection wavelength of 205 nm, injection volume of 10 μL, and column temperature maintained at 37°C. A gradient elution program was employed as follows: 0-19.0 min, 90.5% B; 19.0-25.0 min, 90.5-100% B; 25.0-55.0 min, 100% B. The chromatogram of the saponification solution obtained under these chromatographic conditions is shown in Fig. S16.

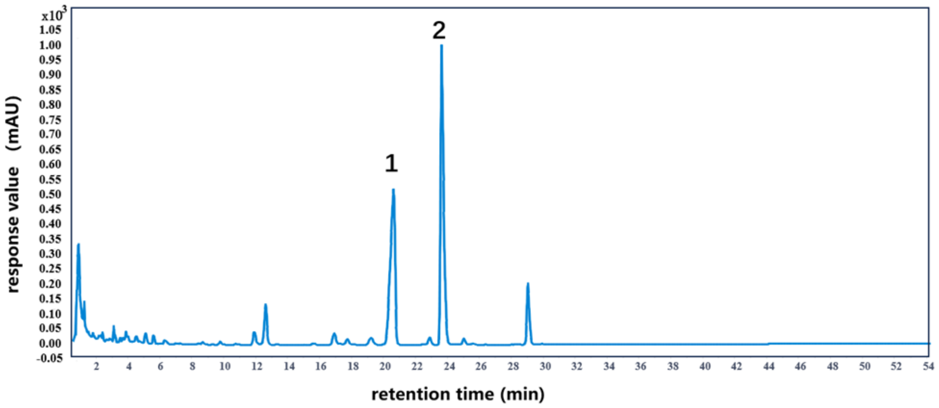

Fig. S16. HPLC of saponification solution (1: cholesterol; 2: lanosterol)

S5 Calculation of cholesterol and lanosterol yield

To facilitate the quantification of lanosterol and cholesterol yields during continuous saponification processes, a calibration curve correlating peak area ratios with lanosterol yield was established. Samples were collected at different time points from batch experiments for this purpose.

Fig. S17 shows the relationship between the lanosterol/cholesterol peak area ratio and the lanosterol/cholesterol yield. The calculation formula for lanosterol yield is given in Equation S1.

$$Y_{LANOSTEROL} = 0.7364 \times R_A - 0.0176 \qquad (S1)$$

where $Y_{LANOSTEROL}$ represents the yield of lanosterol, and R_A represents the peak area ratio of lanosterol to cholesterol. The lanosterol/cholesterol peak area ratio showed excellent linear correlation with lanosterol saponification rate with a coefficient of determination (R^2) exceeding 0.999.

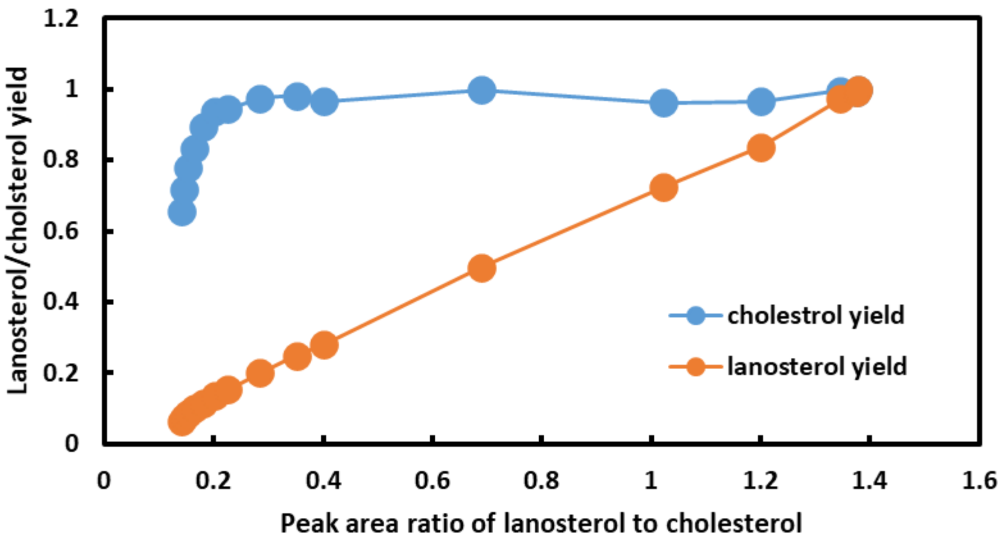

Fig. S17 The relationship between the lanosterol/cholesterol peak area ratio and the lanosterol/cholesterol yield.

For the yield of cholesterol, it rapidly increased and stabilized during the saponification process. When the lanosterol/cholesterol peak area ratio exceeded 0.35, the cholesterol yield reached 100%.
